# Hydrogels and Aerogels for Versatile Photo-/Electro-Chemical and Energy-Related Applications

**DOI:** 10.3390/molecules29163883

**Published:** 2024-08-16

**Authors:** Jiana Sun, Taigang Luo, Mengmeng Zhao, Lin Zhang, Zhengping Zhao, Tao Yu, Yibo Yan

**Affiliations:** 1 Frontiers Science Center for Flexible Electronics (FSCFE), Xi’an Institute of Flexible Electronics (IFE), Xi’an Institute of Biomedical Materials & Engineering (IBME), Northwestern Polytechnical University, 127 West Youyi Road, Xi’an 710072, Chinaiamtyu@nwpu.edu.cn (T.Y.); 2 Zhijiang College, Zhejiang University of Technology, Hangzhou 310014, China

**Keywords:** hydrogel, carbon aerogel, cellulose aerogel, oxide aerogel, flexible sensors, battery, supercapacitor

## Abstract

The development of photo-/electro-chemical and flexible electronics has stimulated research in catalysis, informatics, biomedicine, energy conversion, and storage applications. Gels (e.g., aerogel, hydrogel) comprise a range of polymers with three-dimensional (3D) network structures, where hydrophilic polyacrylamide, polyvinyl alcohol, copolymers, and hydroxides are the most widely studied for hydrogels, whereas 3D graphene, carbon, organic, and inorganic networks are widely studied for aerogels. Encapsulation of functional species with hydrogel building blocks can modify the optoelectronic, physicochemical, and mechanical properties. In addition, aerogels are a set of nanoporous or microporous 3D networks that bridge the macro- and nano-world. Different architectures modulate properties and have been adopted as a backbone substrate, enriching active sites and surface areas for photo-/electro-chemical energy conversion and storage applications. Fabrication via sol–gel processes, module assembly, and template routes have responded to professionalized features and enhanced performance. This review presents the most studied hydrogel materials, the classification of aerogel materials, and their applications in flexible sensors, batteries, supercapacitors, catalysis, biomedical, thermal insulation, etc.

## 1. Introduction

**Figure 1:**
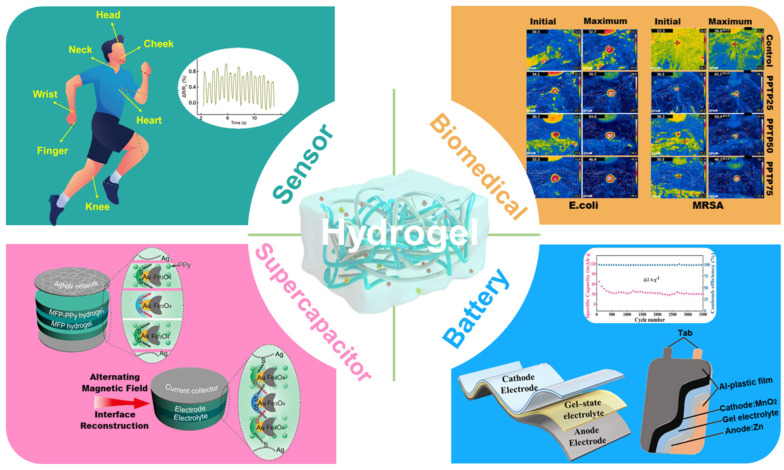
Scheme of hydrogel applications, reprinted with permission [1,2,6,8,9,12], copyright © 2023 John Wiley & Sons, © 2021−2024 Springer Nature and © 2022 Elsevier B.V.

**Figure 2:**
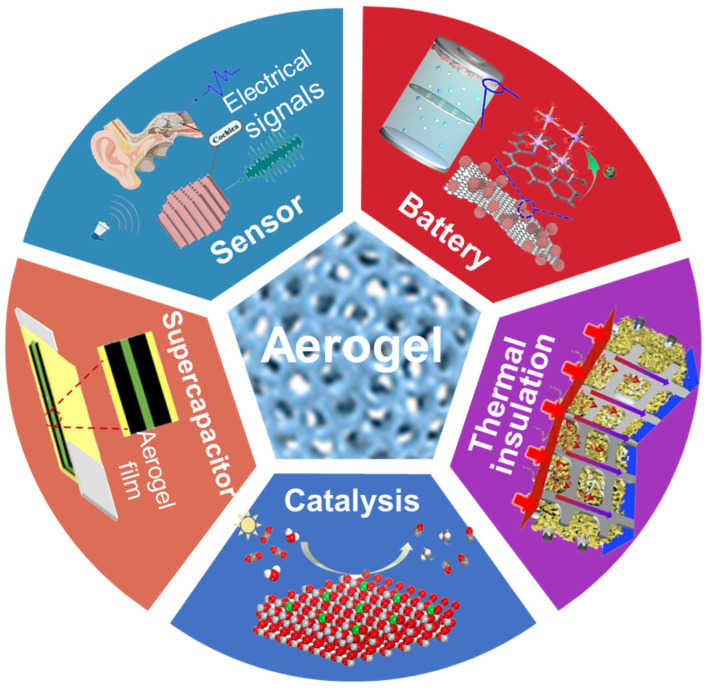
Scheme of aerogel applications, reprinted with permission [3,4,10,11,13,14], copyright © 2019–2023 American Chemical Society, © 2023 Springer Nature, and © 2023 Elsevier B.V.

Tremendous attention has been focused on the development of photo-/electro-chemical and energy-related applications, leading to increasing function modulation of these devices and materials. Aerogels and hydrogels have been frequently studied and used for flexible devices and functional materials, such as sensors [1,2,3], supercapacitors [4,5,6], batteries [7,8,9,10], photo-/electro-catalysis [11], and functional materials [12,13,14] (Figure 1 and Figure 2). Various modifications of 3D networks were applied to create versatile functionalities and enhance performances and stability to adapt to practical applications [15,16,17]. Synthetic methods have evolved from conventional molecule growth to function module assembly. The applications varied from biomedical, catalysis, sensors, supercapacitors, to batteries. Herein, we open discussion with the development from hydrogel to aerogel architectures and then summarize the recent advances in design and production techniques. Prominent applications in photo-/electro-chemical and energy-related applications have been discussed. Subsequently, promising prospects, challenges, and evaluations are proposed in these fields. This review may offer interesting direction and curious inspiration for hydrogels and aerogels in future explorations.

Hydrogels are hydrophilic polymers with 3D networks [18], usually designed and synthesized by polymers and fillers to attain excellent properties. The matrix material is always chosen from polyacrylamide (PAAm), polyvinyl alcohol (PVA), and copolymer, with fillers of polymer particles, nanomaterials, organic and inorganic compounds, etc. Based on its accessibility, biocompatibility, and biodegradability [19], for example, PAAm derivatives are among the most popular matrix materials via free radical crosslinking polymerization solutions [20], hybridized with hydrophilic polysaccharides to promote biocompatible hydrogels [21]. Yet hydrogels easily dehydrate with poor stability and a short lifetime when they are applied; thus, more studies focus on increasing water-retention capacity, hybridizing CaCl_2_ superabsorbent, polydimethylsiloxane (PDMS) [22], and constructing elastomers to optimize the performance and stability.

Aerogels possess interesting characteristics and are regarded as an outstanding substrate in many fields. They are usually produced by sol–gel methods and form solvent-containing networks, then volatilize gels through various routes to modulate porosity structure. In addition, removal of certain components from alloys or composites also evolves aerogels. The category includes carbon aerogel [23,24], organic [25,26,27], and inorganic aerogels [28,29] utilized for sensor, catalysis, battery, supercapacitor, and thermal insulation fields.

Hereby, this work provides a general categorization of aerogels and hydrogels accompanied by their contributions to photo-/electro-chemical catalysis, sensors, batteries, supercapacitors, biomedical, and thermal insulation applications. In recent years, the excellent properties of synthetic hydrogels and aerogels have promoted their applications in flexible equipment and industry.

## 2. Hydrogels

### 2.1. PAAm-Based Hydrogels

**Figure 3:**
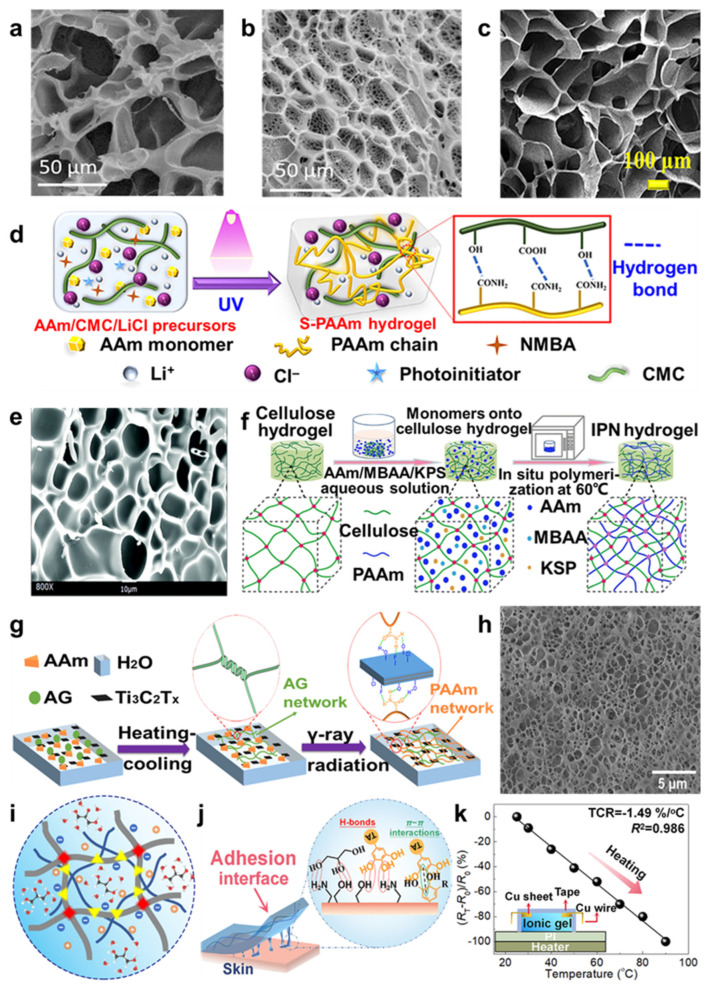
SEM images of (**a**) PAAm/CS hydrogel, (**b**) microgel@PAAm/CS hydrogel, (**c**) S−PAAm hydrogel, (**e**) IPN PAAm−PAAc hydrogel (after freeze−drying), and (**h**) AG/T−PAAm DN hydrogel. Scheme for (**d**) production of S−PAAm hydrogel, (**f**) synthesis process of C−PAAm IPN hydrogels, (**g**) preparation of AG/T−PAAm DN hydrogel, (**i**) structure of PCTN organo−hydrogel. (**j**) Adhesion mechanism of the PCTN used as ionic skin. (**k**) The relative resistance of organo-hydrogel thermistors varied from 25 °C to 90 °C, reprinted with permission [33,34,36,37,41,42], copyright © 2021−2023 American Chemical Society, © 2023 John Wiley & Sons, © 2018 Royal Society of Chemistry, © 2019 Springer Nature, and © 2022 Elsevier B.V., respectively.

Based on the advantages of PAAm polymer, PAAm usually serves as the substrate material for hydrogels. However, pure PAAm hydrogel has poor mechanical properties [30], leading to low utility, so many studies have been carried out to improve its performance by adding fillers to PAAm hydrogel. In recent years, research on PAAm hydrogel has often used the principle of free radical polymerization and added fillers to form semi-interpenetrating polymer networks (semi-IPNs), interpenetrating polymer networks (IPNs), or dual networks (DNs) to improve the properties of PAAm-based hydrogel. The semi-IPN polymer network is usually formed by inserting the chains of a hydrophilic polymer into the network of the crosslinked polymer instead of chemical bonds between polymers, which can combine the properties of polymers and enhance their mechanical strength [31]. For example, Liu et al. [32] synthesized a semi-IPN hydrogel using PAAm and chitosan (CS) via free radical crosslinking polymerization, which showed excellent humic acid adsorption capacity with increasing temperature and can be applied in a wide pH range. The presence of the semi-interpenetrating polymer network formed large pore diameters and strong pore walls, which can improve the mechanical and stable properties. Similarly, Peng et al. [33] also synthesized a PAAm/CS semi-interpenetrating polymer network hybridized with poly(N-isopropylacrylamide-co-acrylic acid) (PNIPAAm-co-AAc)-based microgel particles (microgel@PAAm/CS), forming a pH and temperature dual-responsive hydrogel structure (Figure 3a,b). Moreover, Lu et al. [34] designed the S-PAAm hydrogel with a semi-IPN network, which was fabricated by sodium carboxymethyl cellulose (CMC), lithium chloride (LiCl), and PAAm (Figure 3c,d), and used it to form a sandwich structure with silane-modified polydimethylsiloxane (S-PDMS) elastomers for mechanical–thermal multimode sensing.

The IPN polymer network is produced by combining different pre-polymerized polymer solutions that are partially interconnected with each other on a polymer scale instead of covalently bonded and exhibiting strong mechanical properties [31]. For example, Lee et al. [35] developed a PAAm/gelatin (PG) IPN hydrogel with excellent mechanical strength, self-healing abilities, and strong tissue adhesion, applied as an artificial vocal fold tissue. And Xu et al. [36] also formed an IPN hydrogel between PAAm and poly(acrylic acid) (PAAc) polymer (IPN PAAm-PAAc) (Figure 3e), and Lin et al. [37] fabricated a cellulose–polyacrylamide interpenetrating network (C-PAAm IPN) hydrogel via the in situ polymerization method (Figure 3f); their mechanical properties improved more than a single polymer. Compared to traditional hydrogels, IPN hydrogels show a greater degree of mechanical properties, especially hydrogen bonding, which plays a significant role in their excellent performances and facilitates the energy dissipation of hydrogel networks by breaking hydrogel bonding.

The dual network is fabricated by two different network structures, which can cover their shortages and form a high crosslinking density and mechanical strength hydrogel structure [38]. For instance, Yan et al. [39] prepared gelatin/polyacrylamide/graphene oxide nanocomposite double-network hydrogels (Gelatin/PAAm/GO NC-DN gels), and Feng et al. [40] fabricated polyacrylamide/halloysite nanotubes (PAAm/HNT) nanocomposite hydrogel via in situ free radical polymerization. And the PAAm/HNT hydrogel showed superior conductivity and mechanical properties, which were attributed to the ionic transport, and it can be used to apply a strain sensor to monitor the movement of fingers, knees, etc. In addition, Lin et al. [41] designed a novel agarose/Ti_3_C_2_T_x_-polyacrylamide (AG/T-PAAm) DN hydrogel, which was fabricated via heating–cooling and γ-ray radiation-induced polymerization (Figure 3g,h). The AG/T-PAM DN hydrogel exhibited excellent mechanical properties owing to the conductive double-network structure within the hydrogel, superior adhesion, and high sensitivity, and was expected to be applied for strain sensor monitoring the various movements of humans.

In order to improve the mechanical properties by adding fillers to the PAAm-based hydrogel to form a network structure, there are also some studies focusing on giving the PAAm-based hydrogel more properties to meet the needs of multiple applications. For example, Wei et al. [42] developed a novel strategy to fabricate a multifunctional organo-hydrogel, introducing cellulose nanofibrils (CNF), tannic acid (TA), conductive ions (NaCl), and glycerol–water solvent into PAAm hydrogels (PCTN) through one-step UV-initiated polymerization (Figure 3i), where PAAm served as the matrix material, the CNF served as a rigid backbone to enhance the mechanical strength and increase the ionic conductivity, and the TA component had abundant catechol groups providing the adhesive and intimate interfacial contact. Therefore, the multifunctional organo-hydrogel could serve as the ionic skin for intelligent skin-like devices, monitoring movement and dynamic temperature variation (Figure 3j,k). According to the versatile structure–function exploitation of organo-hydrogel, it could provide great assistance for the development of the next generation of intelligent electronic products.

### 2.2. PVA-Based Hydrogels

**Figure 4:**
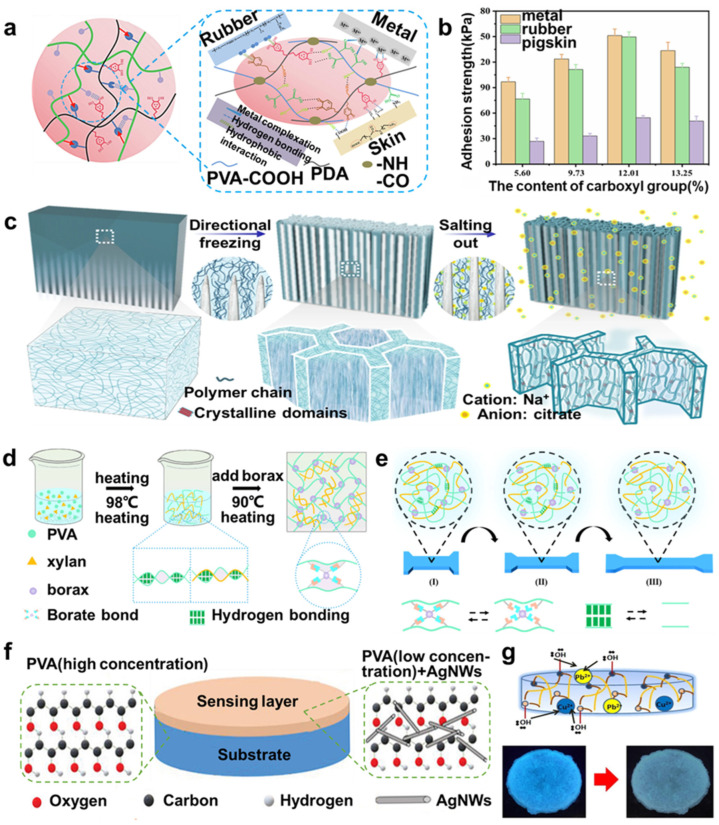
(**a**) Scheme of PVA-COOH/PDA hydrogel structure. (**b**) Adhesion strength diagram of PVA-COOH/PDA hydrogels with different carboxyl group contents on different materials. (**c**) Scheme of fabrication procedure for HA-PVA hydrogels. (**d**) Scheme of the fabrication of xylan/PVA/B DN hydrogel. (**e**) Diagram of bond-breaking changes of xylan/PVA/B DN hydrogel under tension. (**f**) Scheme of the structure of biocompatible hydrogel sensor. (**g**) The mechanism of copper and lead ion detection and removal, reprinted with permission [21,43,44,45,47], copyright © 2020–2021 Elsevier B.V., © 2021 Springer Nature, and © 2020 John Wiley & Sons., respectively.

Similarly, PVA has also been frequently used as a substrate for hydrogels. For example, Yu et al. [43] designed and developed a highly self-adhesive and conductive PVA-based hydrogel by introducing carboxyl groups (-COOH) and a crosslinking agent (polydopamine, PDA) into PVA (Figure 4a). Then, they attained a PVA-COOH/PDA hydrogel, exhibiting high mechanical and self-adhesion properties (Figure 4b), due to the introduction of PDA, which increased the density of the network and decreased the mobility of the molecular chain, and the carboxyl group offered more interaction sites on the surface of the hydrogel.

In order to improve the performance of PVA-based hydrogel, many studies make use of toughening mechanisms to synthesize PVA-based hydrogel, such as double-network structure, nanocomposite structure, etc. For example, Hua et al. [44] designed a PVA hydrogel with hierarchical and anisotropic structure (HA-PVA hydrogel) by freezing-cast and salting-out treatment, consisting of micrometer-scale honeycomb pore walls, which in turn are composed of interconnected nanofibrous networks. (Figure 4c) Based on the unique synergy of the freezing-assisted salting-out method, which boosted the effect of aggregation, the hydrogels exhibited strong, tough, stretchable, and fatigue-resistant properties. The strengthening mechanism of designed hydrogels was attributed to the formation of hydrogen bonds and crystal domains leading to structure densification, and the toughening mechanism was mainly the fibril pulling out, bridging, and energy dissipating. And the designed hydrogel structure was expected to be applied to the medical, robotics, energy, and additive manufacturing fields. And Ai et al. [21] fabricated a double-network hydrogel using PVA-borax self-healing hydrogels and xylan (xylan/PVA/B DN hydrogel); the double-network structure was composed of numerous hydrogen bonds between xylan and PVA and the generation of the PVA crystal domain after a freeze-thaw process (Figure 4d,e). The hydrogel demonstrated excellent mechanical performance, self-healing ability, and pressure remodeling properties for various applications.

Adding nanocomposite structure is another way to improve the properties of PVA-based hydrogels and apply them in more fields. For example, Azadi et al. [45] presented a biocompatible hydrogel sensor constituted of PVA and silver nanowires (AgNWs), forming a nanocomposite structure (Figure 4f). The freeze–thaw method was employed in this study, and the results proved that using this method to fabricate crosslinking structures could produce stable hydrogels without extra crosslinking agents. And adding the AgNWs in the sensing layer endowed the hydrogel with excellent biocompatibility, high sensitivity, and mechanical performance, which were beneficial to applying in flexible wearable fields. Similarly, Singh et al. [46] fabricated a PVA-based hydrogel that was composed of copolymerizing PVA and silver nanoparticles with biogenic nanostructured materials, xanthan gum (XG), hydroxyl propyl methyl cellulose (HPMC), and sodium carboxymethyl cellulose (NaCMC), respectively, exhibiting superior antibacterial and biocompatible properties for wound healing fields. What is more, many studies attach importance to designing PVA-based hydrogel films so that they can be applied to more fields. For example, Sharma et al. [47] designed a quinine derivative of dextrin/PVA-based hybrid gel film via enzymatic construction for detecting and removing copper and lead ions in real water samples (Figure 4g). Hydrogels were commonly used for removing impurities, and the new derived hybrid hydrogel could first selectively detect copper and lead ions and further remove them.

### 2.3. Copolymer-Based Hydrogels

**Figure 5:**
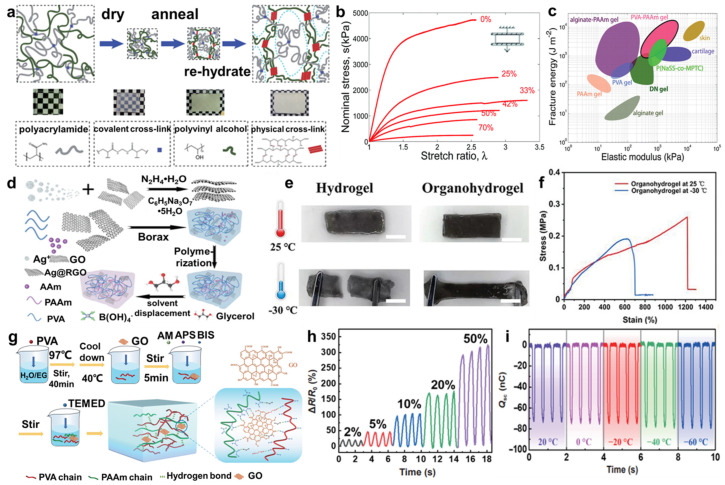
(**a**) Scheme of the production process and structure of the hybrid hydrogel. (**b**) Stress–stretch curves of hybrid hydrogels of various weight percentages of acrylamide as labeled. (**c**) Chart for fracture energy versus elastic modulus compared with various soft materials. (**d**) Scheme of the synthetic process and structure of the Ag@rGO/PVA−PAAm hydrogel. (**e**) Self-healing and (**f**) anti-freezing properties of Ag@rGO/PVA−PAAm hydrogel contrasted with hydrogel at 25 and −30 °C. (**g**) Schematic diagram of fabrication of GPPD-hydrogel. (**h**) Strain sensing performance for different tensile strain GPPD−hydrogel. (**i**) The output performance of GPPD−TENG at different temperatures at frequency of 2 Hz, reprinted with permission [48,49,50], copyright © 2014 Royal Society of Chemistry, © 2022 John Wiley & Sons, and © 2022 Springer Nature, respectively.

A copolymer usually consists of a composite of two or more polymers, and more studies have concentrated on using it as the composition of hydrogels, especially PVA and PAAm composite materials. For example, Li et al. [48] proposed that a hybrid network of a crystalline polymer and a covalently crosslinked hydrophilic polymer formed a hydrogel with robust mechanical properties and good chemical stability. They used PVA as the crystalline polymer and PAAm as the covalently crosslinked hydrophilic polymer to synthesize PVA-PAAm hydrogel (Figure 5a), exhibiting high stiffness, strength, and toughness (Figure 5b,c). Furthermore, Zhang et al. [49] introduced Ag nanoparticles and reduced graphene oxide sheets into PVA-PAAm dual network hydrogel (Figure 5d), showing anti-freezing and self-healing properties (Figure 5e,f), and Dai et al. [50] combined graphene oxide with PVA-PAAm bi-network hydrogel (GPPD-hydrogel) (Figure 5g), exhibiting highly stretchable, conductive, and frost resistance (Figure 5h,i). In addition, novel hybridization structures such as metal particles [51], NaCl [52], nanomaterials [53], polydopamine, and the tetrapeptide Arg-Glu-Asp-Val [54] are for the design of multifunctional materials with improved performance.

### 2.4. Graphene Hydrogels

Graphene hydrogels combine the advantages of graphene (such as high conductivity, good thermal conductivity, etc.) and the properties of hydrogel (such as flexibility, hydrophilicity, etc.), which affords broad application prospects. The laser-induced graphene (LIG) hybridized composite of polyvinyl alcohol, phytic acid, and honey (PPH) hydrogel exhibited the characteristics of good Young’s modulus, viscosity, and antibacterial properties [55]. Compared with LIG on PDMS films, the inherent tensile strength of conductive nanocomposites has been improved by more than five times. Using PPH hydrogel as the energy dissipation interface and the circuit path out of the plane, continuous deflection cracks can be induced in LIG, and the tensile capacity can be expanded from 20% to ~110%. The saturation tensile strain of approximately 220% was achieved via further modulation. Moreover, the resistance of nanocomposites exhibits a positive linear output with tensile strain, providing a potential pathway for decoupling the effects of strain on different stimuli. Accordingly, stretchable skin sensors were prepared with excellent detection of mechanical, temperature, humidity, and electrocardiogram sensing, as well as heart activity monitoring in vivo. In addition, combining biomimetic graphene microtubule with poly(N-isopropylacrylamide) (PNIPAM) hydrogel at a porosity of 5.4% significantly improved the actuation kinetics by ~400% and the actuation stress by ~4000% without losing mechanical stability, overwhelming the limitations of water transport [56]. Graphene microtubules enable unconstrained optical control and electric actuation. It is expected to inspire a multifunctional platform for improving the functionality of soft matter via joining responsiveness and 2D materials, driving the development of intelligent soft sensors and actuators.

## 3. Aerogels

### 3.1. Carbon Aerogels

#### 3.1.1. Resorcinol Formaldehyde (RF) Aerogels

**Figure 6:**
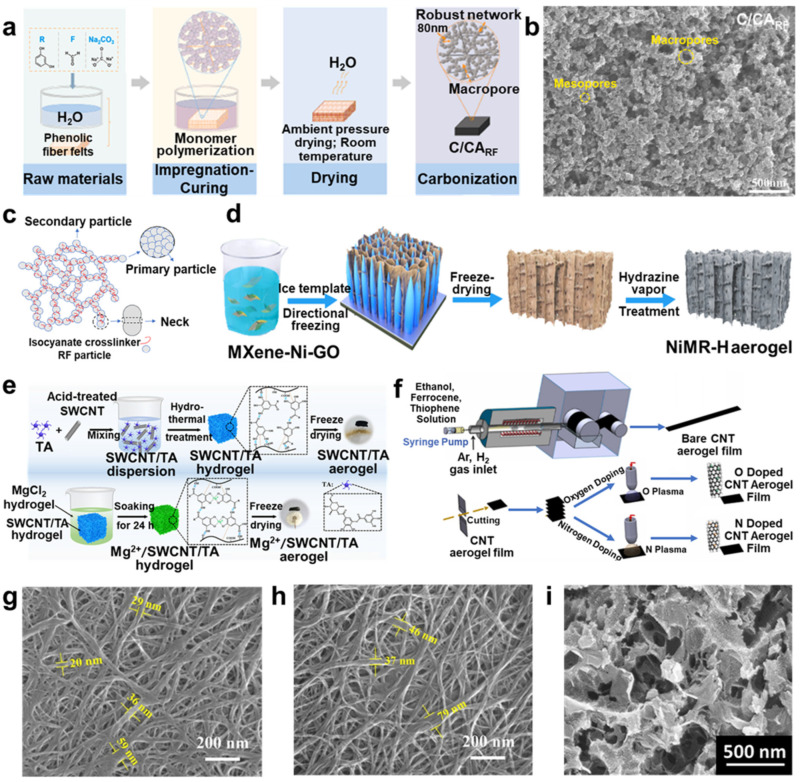
(**a**) Scheme of the C/CA composite production using RF aerogel as precursor. (**b**) SEM image of the C/CA_RF_ at carbonized state. (**c**) Schematic of the crosslinked RF aerogel structure. (**d**) Schematic illustration of the fabrication process of a NiMR-H aerogel. (**e**) Scheme for SWCNT/TA and Mg^2+^/SWCNT/TA aerogel production. (**f**) Scheme of the fabrication process of O− and N−doped CNT aerogel film. SEM images of (**g**) SWCNT/TA, (**h**) Mg^2+^/SWCNT/TA, and (**i**) N−doped carbon aerogels, with permissions [16,60,61,67,68,69], copyright © 2015−2023 Elsevier B.V., © 2019 John Wiley & Sons, and © 2021–2023 American Chemical Society, respectively.

RF aerogels are usually prepared by the aqueous polycondensation of resorcinol formaldehydes and dried in specific ways in certain environments. There are many studies focusing on the conditions of drying for carbon aerogels, and drying methods mainly include freeze drying, supercritical drying, and ambient drying [57]. Drying environments usually use acetone [58] and CO_2_ [59]. For instance, Zhang et al. [60] fabricated carbon fiber-reinforced carbon aerogel (C/CA) composites using RF aerogels as precursors (C/CA_RF_) (Figure 6a,b). Supercritical drying is widely used for RF aerogels because it can keep the shape of the gels and form ultrafine porosity structures.

To enhance mechanical properties, lots of studies have explored various methods for reinforcement of structures to reduce brittleness by using crosslinking agents or adding additives. For example, Aghabararpour et al. [61] used two crosslinkers, hexamethylene diisocyanate (HDI) and methylene diphenyl diisocyanate (MDI), to improve mechanical properties, and they discovered that increasing the content of crosslinkers can obtain a higher improvement in mechanical properties (Figure 6c). Moreover, many kinds of additives have been applied, such as graphene oxide [62], co-assembled polyacrylonitrile nanofibers and graphene oxide nanosheets [63], etc.

#### 3.1.2. Carbon Nanotube (CNT) Aerogel

CNT is a one-dimensional nanomaterial with a special architecture and light weight, showing excellent mechanical, electrical, and physicochemical properties [64]. Therefore, numerous researchers used it to prepare CNT aerogel, which was porous with an isotropic structure, causing low mechanical strength in the final materials. Hence, many strategies have been applied to enhance the properties, such as elemental doping [65], hybridizing nanoparticles [66], and functionalization. Especially for elemental doping, which was frequently studied and applied. For example, Liao et al. [67] designed an ultralight, biocompatible, and conductive aerogel composed of tannic acid (TA), single-walled carbon nanotubes (SWCNTs), and MgCl_2_ (Mg^2+^) (Figure 6e). All aerogels possess an interconnected layered porous framework of coiled carbon nanotube sheets with continuous large pores (Figure 6g,h), and the aerogel can be applied for flexible pressure sensors and monitors. In addition, Prakash et al. [16] doped O/N into CNT aerogel film using plasma treatment by an in situ self-assembly process and used a chemical vapor deposition process to fabricate electrodes (Figure 6f). Then, the CNT aerogel film showed excellent mechanical stability, repeatability, and sensitivity applied to cost-efficient volatile organic compound detection. 

#### 3.1.3. Graphene Aerogel

Graphene is a two-dimensional carbon material; carbon atoms connected by sp^2^ hybrids are packed tightly together, exhibiting similar properties to CNT. Therefore, it is considered a candidate for supercapacitors and electrode materials.

In recent years, many studies have focused on endowing it with more properties to better apply in more fields. One of the ways is elemental doping to enhance its electronic properties to better apply in supercapacitors. For example, Hao et al. [68] used chitosan to fabricate graphene-based nitrogen-self-doped hierarchical porous carbon aerogels (Figure 6i). The synthesized graphene aerogels used this method, showing high specific capacitance and outstanding cyclability, and they were suitable to apply in supercapacitors. Except for this application, Liang et al. [69] designed a 3D dielectric/magnetic multicomponent Ni/MXene/RGO (NiMR-H) aerogel using graphene oxide (GO), Ti_3_C_2_T_x_ MXene, and Ni nanochains by directional freezing method and hydrazine vapor treatment (Figure 6d). The aerogel exhibited outstanding electromagnetic wave absorption performance and had a wide range of applications in various environments.

### 3.2. Organic Aerogels

**Figure 7:**
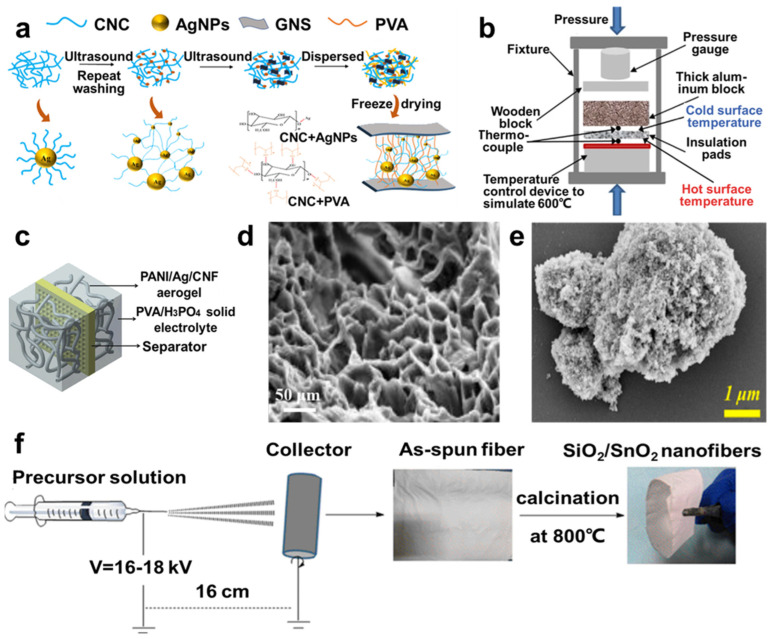
(**a**) Scheme of the fabrication process of PVA/CNC/GNS composite aerogel. (**b**) Thermal insulation scheme of fiber-reinforced SiO_2_ aerogel thermal insulation pads. (**c**) Fabrication of polyaniline/silver/cellulose nanofibril aerogel supercapacitors. SEM images of (**d**) PVA/CNC/GNS composite aerogel and (**e**) ZrO_2_–Al_2_O_3_ composite aerogel coating. (**f**) Electrospinning fabrication processes of SiO_2_/SnO_2_ nanofibers, reprinted with permissions [71,72,74,75,76], copyright © 2014–2022 Elsevier B.V., © 2023 Springer Nature, and © 2019 The American Ceramic Society, respectively.

Organic aerogels are prepared by sol–gel methods and removing solvent through drying methods, which are similar to the drying methods of carbon aerogels [70]. Then, different methods are used to synthesize different organic aerogels, depending on the requirements of their properties and applications. For example, cellulose aerogels can be classified into three categories [27], including cellulose nanocrystals (CNCs), cellulose nanofibers (CNFs), and bacterial cellulose (BC). CNCs were prepared by the concentrated acid hydrolysis of amorphous regions with rigid rod nanostructures. CNFs are irregular entangling fibers with a 3D network structure, showing excellent flexibility and fitting for aerogel structures. BCs are fiber bundles consisting of several kinds of microorganisms, with a subtle network and excellent mechanical stability [25]. Multiple studies concentrated on constructing new structures for various applications. For example, Zhang et al. [71] prepared a novel 3D crosslinked structure by using cellulose nanocrystals, graphene nanosheets, and polyvinyl alcohol (Figure 7a,d), fabricating a composite aerogel with high strength, super toughness, stretchability, and antibacterial properties so it can be applied to detect human movements in flexible strain sensors. Moreover, some research on aerogels focused on applying them for supercapacitors electrode materials; for example, Zhang et al. [72] designed and synthesized polyaniline/Ag/cellulose (PANI/Ag/CNF) aerogel (Figure 7c), and Niu et al. [73] formed polypyrrole/cellulose nanofiber aerogel, which can be employed for supercapacitors.

### 3.3. Oxide Aerogels

#### 3.3.1. SiO_2_ Aerogels

SiO_2_ aerogels have excellent performance, such as high specific surface area, low density, and low thermal conductivity for thermal insulation [77]. Based on their properties, Wang et al. [74] prepared SiO_2_ aerogels by using the supercritical ethanol (SCE) drying technique and using fiber to reinforce SiO_2_ aerogels. And using products such as a fabricated cold retaining blanket and a lithium battery insulation sheet, after a series of tests, it showed benign cold and heat insulation properties (Figure 7b), providing high potential for thermal insulation. Moreover, Zhang et al. [75] designed SiO_2_/SnO_2_ nanofiber (SSNF)-reinforced SiO_2_ (SiO_2_-SSNF) aerogel composites through electrospinning technology and SCE drying technique (Figure 7f), and Shen et al. [78] fabricated a carbon nanotube silica (CNTs-SiO_2_) aerogel hybrid as the superhydrophobic coating, exhibiting enhanced thermal insulation and mechanical properties.

#### 3.3.2. Al_2_O_3_ Aerogels

Based on some studies about metal oxide aerogels, metal oxide aerogels are usually considered distinguished thermal insulation materials [79], but they may undergo degradation at high temperatures. Al_2_O_3_ aerogels may degrade microstructure over 1000 °C [80]. When using Al_2_O_3_ aerogels at high temperatures, composite aerogel coating was frequently applied to more areas. For example, Gao et al. [76] designed new methods by introducing aluminum dihydrogen phosphate as a high-temperature adhesive and aluminum isopropoxide (C_9_H_21_AlO_3_) as a reducing agent into ZrO_2_–Al_2_O_3_ composite aerogel, and the composite aerogel coating, whose structure presented independent flake or block form without tight adhesion to each other (Figure 7e), exhibited high-temperature resistance, flame retardance, stability, and rapid heat dissipation.

## 4. Applications

### 4.1. Sensors

In the era of intelligent information, precisely collecting real-time information is essential and necessary, and sensors play an important role in obtaining information. Moreover, many studies have focused on using hydrogels, polydimethylsiloxane elastomers, and aerogels to fabricate flexible and intelligent sensors.

**Figure 8:**
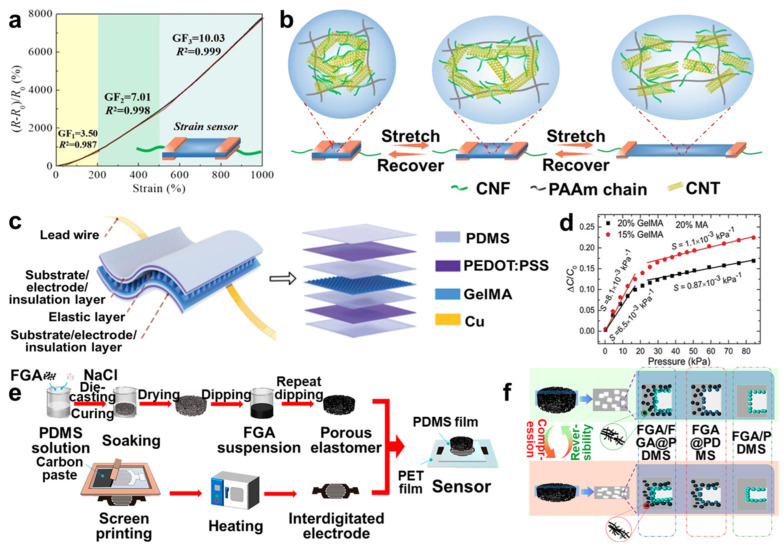
(**a**) The strain response curves of the PCCG organo−hydrogel. (**b**) The conductive network change procedure in the PCCG organo−hydrogel upon stretching. (**c**) Schematic diagram of GelMA hydrogel−based pressure sensor structure. (**d**) The variation in pressure sensitivity as a function of GelMA concentrations. (**e**) The fabrication procedure of the FGA/FGA@PDMS pressure sensor. (**f**) The mechanism of FGA/FGA@PDMS flexible pressure sensor, reprinted with permission [86,88,90], copyright © 2022 Springer Nature, © 2020 John Wiley & Sons, and © 2023 American Chemical Society, respectively.

Hydrogels and aerogels have special mechanical flexibility and durability; hence, they are promising candidates for strain sensors [81,82], pressure sensors [83,84], gas sensors [66], and various intelligent detectors [42]. For example, Wei et al. [85] combined freezing and salting out to fabricate PVA hydrogel hybridized graphene oxide (GO) to form a PVA/GO network, then freezing and soaking in an aqueous NaCl solution. This method improved mechanical strength and conductivity by forming channels in the hydrogel network to transport Na^+^ and Cl^−^. The PVA/GO strain sensor showed excellent sensitivity when it was used to respond to the real-time bending motion of the wrist and fingers. Moreover, conductive organo-hydrogel was synthesized using cellulose nanofiber/carbon nanotube (CNF/CNT) nanohybrids and polyacrylamide-glycerol (PAAm-Gly), namely PCCG organo-hydrogel, which exhibited benign conductivity vs. mechanical properties [86]. When it was used as a strain sensor, the dynamic rearrangement of the CNF/CNT nanohybrids in the polymer matrix demonstrated remarkable strain responsiveness (Figure 8a,b). Hence, introducing nanohybrids into hydrogels is a suitable approach to enhance sensitivities and mechanical properties for sensors.

In addition, hydrogels and aerogels are applicable for pressure sensors extending to multifunctional fields. For example, Jiang et al. [87] designed conductive hydrogels by doping various conductive materials, tannic acid-modified boron nitride nanosheets (BNNS-TA), and Fe^3+^ ions into hydrogels and preparing a BNNS-TA/Fe^3+^/PAAm hydrogel. Based on the combination of multiple intermolecular interactions, such as covalent crosslinking, ion coordination, and hydrogen bonding, the ternary BNNS-TA/Fe^3+^/PAAm hydrogel exhibited excellent pressure-sensing performance, compressive strength, and recovery ability. Upon assembly into wearable pressure sensors, it could detect diverse human activities. Similarly, Li et al. [88] designed a solution-processable and transparent gelatin methacryloyl (GelMA) hydrogel pressure sensor, showing adjustable pressure sensitivity with high sensitivity and a low detection limit (Figure 8c,d). Moreover, Sun et al. [89] combined cellulose nanocrystals/poly(ethylene glycol) aerogel with PAAm elastomer, fabricating a bio-based pressure-sensitive and color-adjustable structure that exhibited iridescent structure and superior fatigue resistance and was used for pressure sensors, screen displays, etc. In addition, Luo et al. [90] designed a pressure sensor based on fragmented graphene aerogel (FGA)/polydimethylsiloxane (PDMS) sponges (FGA/FGA@PDMS) pressure sensors, presenting higher sensitivity and rapid and stable response (Figure 8e,f). Therefore, the development of multifunctional gel-based sensors has become a general trend and can meet more application demands.

**Figure 9:**
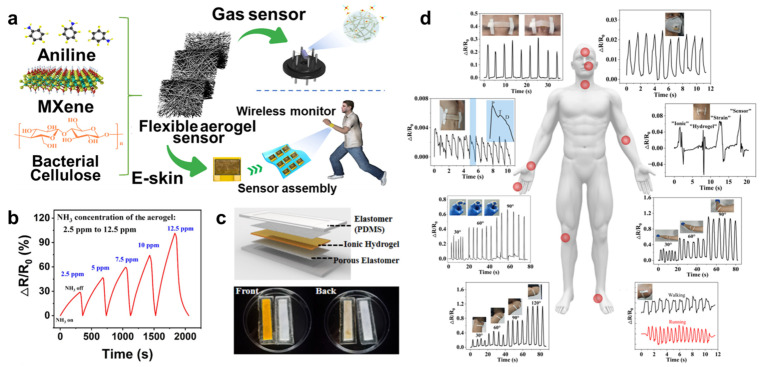
(**a**) Structure of MXene/PANI/BC aerogel for the applications of e-skins and gas sensors. (**b**) Response of the MXene/PANI/BC composite aerogel-based gas sensor of NH_3_ gas. (**c**) Scheme of PDMS-encapsulated ionic hydrogel and PDMS-encapsulated PVA-G-FeCl_3_ and PVA-G hydrogel. (**d**) The PVA-G-FeCl_3_/PDMS hybrid hydrogel for human motion monitoring, reprinted with permission [92,93], copyright © American Chemical Society and © MDPI, respectively.

Furthermore, hydrogels and aerogels can be utilized as gas (e.g., NO_2_, NH_3_) sensors [91]. There were three synergetic supramolecular interactions, hydrogen bonding, molecule crystallization, and electrostatic interactions existing in hydroxyls, PVA crystallization, and poly(ionic liquids) of the intrinsic hydrogel networks for the gas sensor. Zhi et al. [92] designed a MXene/polyaniline/bacterial cellulose (MXene/PANI/BC) aerogel that can be applied for pressure-sensitive NH_3_ gas sensors (Figure 9a,b). These were attributed to the terminal groups of MXene, which can connect actively with target gas molecules, and hydrogen bonds formed in this system. Therefore, the sensor exhibited a rapid and reversible response and good mechanical and recovery properties. The novel sensing mechanism will promote the development of hydrogel hybrid networks for gas sensor applications.

However, hydrogels are mainly composed of water, which easily dehydrates and limits performance or stability. To overcome this problem, encapsulation of hydrogels with a polymer coating to retain water has been developed. PDMS has good physicochemical stability, hydrophobicity, non-toxicity, insulation, and mechanical properties, allowing wide applications in microfluidic chips, packaging, and other fields. For example, Zhao et al. [93] designed a mechanically interlocked hydrogel–elastomer strain sensor; they synthesized a PVA hydrogel with glycerol as a crosslinking agent and FeCl_3_ as an electrolyte (PVA-G-FeCl_3_) using PDMS-encapsulated ionic hydrogel (Figure 9c). The encapsulated ionic hydrogel showed excellent conductivity and anti-dehydration properties at different temperatures when it was used as an excellent conductivity-responsive strain sensor (Figure 9d). Hence, elastomer–hydrogel composites can serve as suitable hybridized systems to improve the water-retention capacity, stability, and overall performance of hydrogel.

**Figure 10:**
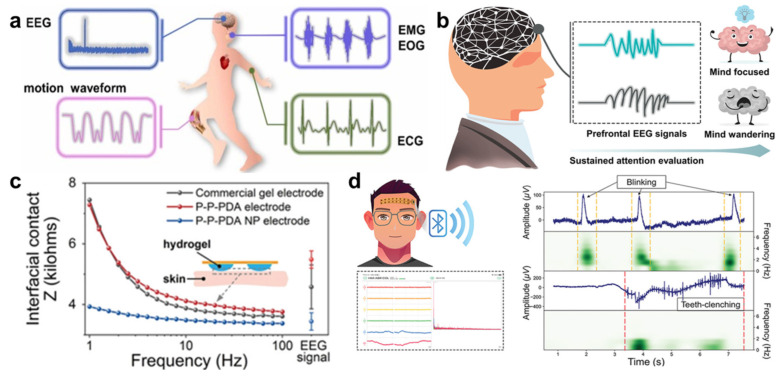
(**a**) Schematic diagram of elastomer−hydrogel integration as skin sensor and flexible electrode for detecting physiological health signal. (**b**) Application of hydrogel electrodes in sustained attention evaluation. (**c**) Interfacial contact impedance of different electrodes under real EEG signals. (**d**) Schematic diagram of the mechanism and evaluation of EEG acquisition system for wireless transmission, reprinted with permission [103,106], copyright © 2022 Elsevier B.V. and © 2023 John Wiley & Sons, respectively.

Furthermore, the hydrogel-based sensors can be applied for recording physiological health signals, such as electroencephalography (EEG) [94,95,96], electrocardiogram (ECG) [97,98,99], electromyography (EMG) [100,101,102], and electrooculogram (EOG) [103,104] (Figure 10a). Especially for EEG electrodes, compared with other electrodes, such as wet electrodes, dry electrodes, and semi-dry electrodes, hydrogel electrodes have the merits of low impedance, excellent adhesion, stability, and biocompatibility for various applications [105]. For example, Han et al. [106] fabricated the network structure of hydrogel via a ketalization reaction of PVA with polyvinylpyrrolidone (PVP) and introduced polydopamine nanoparticles (PDA NPs) via an oxidative degradation process. The hydrogel exhibited conductivity, self-adhesiveness, low modulus, flexibility, and transparency, ascribed to the synergistic effect of nanoparticle enhancement and homogeneous networks. Using the hydrogel to fabricate multichannel hydrogel electrodes could be used for recording EEG signals and real-time transferring EEG signals (Figure 10b–d), promoting the development of EEG monitoring.

**Figure 11:**
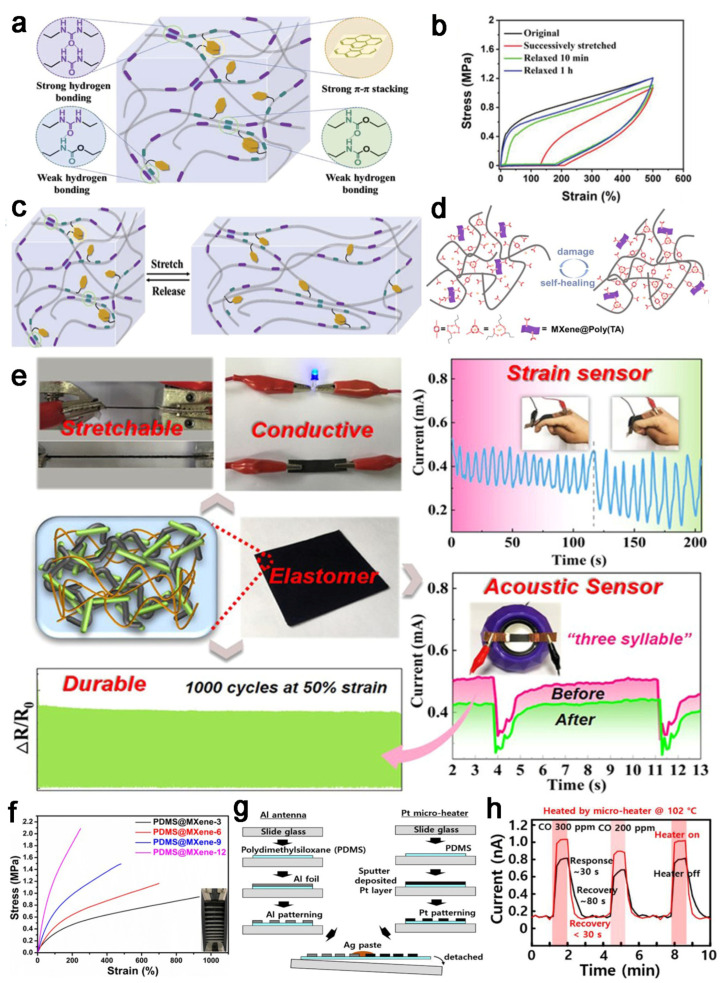
(**a**) Schematic diagram of the dynamic network of healable and recyclable PDMS-based elastomers (H-PDMS-Pym). (**b**) Stress–strain curves of cyclic loading–unloading for H-PDMS-Py0.25 at 500%. (**c**) Schematic diagram cyclic stretching/releasing for sacrificial bond rupture and reshuffling. (**d**) Schematic diagram of self-healing procedure of PDMS@MXene. (**e**) Digital photograph of the SCNC-CNT/PDMS elastomer and schematic illustration of the application of SCNC-CNT/PDMS-based as sensor. (**f**) Stress–strain curves for PDMS@MXene conductive elastomers of modified-MXene with different mass ratios. (**g**) Fabrication of a wireless micro-heater device. (**h**) The relationship of current in SnO_2_ nanowires and CO gas levels in the absence of a heater (black line) and in the presence of a heater (red line), reprinted with permission [111,112,113,115], copyright © 2022 John Wiley & Sons, © 2021 American Chemical Society, © 2022 Elsevier B.V., © 2022 MDPI, respectively.

PDMS is a linear polymer composed of -Si–O–Si- bonds, exhibiting excellent thermal stability, biocompatibility, ductility, and stability [107]. PDMS is widely studied on wearable devices and sensors, where it is used as a substrate and encapsulation component. Compared with hydrogels, PDMS elastomer exhibits superior mechanical properties and long-term durability, whereas hydrogel merely dehydrates during its short lifetime, hindering its further development. Therefore, many researchers focus on strengthening conductivity by adding conductive fillers such as metal nanoparticles [108], conductive polymers [109], and conductive hybrids. For example, Zhang et al. [22] modified PDMS by using conductive hybridized silver nanowires (AgNWs)/graphene, which showed excellent stretchability and sensitivity when applied to strain sensors. To overcome the demerits of low conductivity and other shortcomings, various techniques have been employed to modify the functionalities. For example, Huang et al. [110] changed the structure of PDMS by using a facile solvothermal polymerization process, affording the controllable mechanical properties of the final products. In addition, Liu et al. [111] hybridized different supramolecular interactions, including π–π stacking and hydrogen bonding (Figure 11a), into the matrix via one-pot polycondensation reaction, endowing final elastomers with ultra-healing, highly recyclable, and stretchable performance (Figure 11b,c), promoting their applications in sensors and electronics.

Currently, tremendous studies of PDMS-based elastomers tend to apply to various sensors, such as strain sensors, piezoresistive sensors, and gas detectors. Carbon materials have attracted wide attention as fillers in PDMS-based elastomers. For example, Zhu et al. [112] synthesized a flexible and stretchable conductive PDMS elastomer via using silylated cellulose nanocrystals and carbon nanotubes as fillers, forming the conductive elastomer of SCNC-CNT/PDMS, and the elastomer formed a conductive network affording stretching sensing mechanisms, showing high sensitivity, stretchability, and durability so as to allow outstanding performance of strain sensors (Figure 11e). Similarly, Wang et al. [113] fabricated a PDMS/MXene conductive elastomer based on the reversible interactions, showing self-repairing and stretchable capabilities for strain sensors (Figure 11d,f). PDMS hybridized elastomers also form piezoresistive sensors monitoring the movement of a crane robot [114]. Furthermore, Cho et al. [115] fabricated a wireless thin-film micro-heater on the stretchable PDMS elastomer for wearable gas sensor applications (Figure 11g,h). Thus, PDMS is an ideal matrix substrate through optimizing processing methods and adding fillers to enhance performance, meeting the needs of applications in sensors, electronics, and far more extensive development prospects.

### 4.2. Batteries

In addition to flexible sensors and wearable electronics, energy conversion and storage devices are also urgently demanded, where batteries have drawn wide attention. Growing studies have been focused on improving membrane, electrolyte, and electrode materials, where hydrogels and aerogels can play significant roles in batteries.

#### 4.2.1. Electrolytes

Metal–air batteries, including zinc–air batteries (ZAB) and zinc-ion batteries (ZIB), have shown novel developments in the electrolyte formula, which not only require excellent mechanical properties but also demand superior electro-chemical performance. However, alkaline electrolytes did not satisfy these needs for long-term utility, wearability, or flexibility. Thus, flexible gel electrolytes can solve these problems and show promising application prospects.

**Figure 12:**
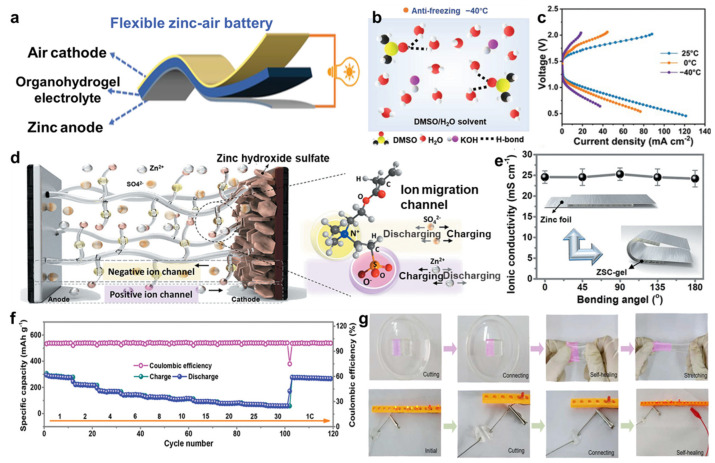
(**a**) Illustration for the structure of flexible zinc−air battery. (**b**) The antifreeze mechanism of OHE. (**c**) Charge/discharge curves of OHE−based flexible ZABs at different temperature. (**d**) Schematic structure of flexible zinc-ion battery with the ZSC−gel electrolyte under an external electric field. (**e**) The curve of ionic conductivity and bending angle of the ZSC−gel electrolyte. (**f**) Cycling performances at different C−rates of the Zn−MnO_2_ coin cells with the ZSC−gel electrolyte. (**g**) Photographs of the self−healing process for Zn^2+^-CS/PAAM hydrogel, reprinted with permission [116,118,119], copyright © 2020–2022 John Wiley & Sons and © 2023 Elsevier B.V., respectively.

Flexible hydrogel electrolytes have drawn great curiosity for ZAB with high ion conductivity and good mechanical performance, which is beneficial to apply in portable or wearable electronics. For instance, Jiang et al. [116] fabricated an organo-hydrogel electrolyte (OHE) with excellent anti-freezing and electro-chemical properties, prepared by immersing poly(2-acrylamido-2-methylpropanesulfonic acid)/polyacrylamide hydrogel (PAMPS/PAAm) in DMSO additive aqueous KOH electrolyte (Figure 12a,b). Adding DMSO to an aqueous KOH electrolyte can form more hydrogen bonding and change the conventional path of the oxygen evolution reaction in ZABs, which then showed long cycling life and superior electro-chemical performances at −40 °C (Figure 12c). Similarly, Liu et al. [117] designed a functional double-network hydrogel electrolyte (FDHE) by fabricating the dual network of PAMPS/PAAm and adding DMSO, which also showed good antifreeze performance. When it was applied in Zn-ion batteries, Zn/PANI@CC batteries showed excellent electro-chemical performances with a specific capacitance of 247.8 mA∙h∙g^−1^ at 3 A∙g^−1^ and the capacity retention attained 96% after 1000 cycles. Except for these, Mo et al. [118] designed a zwitterionic sulfobetaine/cellulose (ZSC) hydrogel electrolyte, which was prepared by natural plants. When ZSC electrolyte was applied to ZIB, it exhibited remarkable electro-chemical stability and mechanical flexibility owing to the particular architecture of nanofibrils (Figure 12d–f). In addition, Liu et al. [119] designed and synthesized a soaking-free and self-healing hydrogel electrolyte for ZIBs (Figure 12g). They introduced the Zn^2+^ ion as a crosslinking agent for CS/PAAm dual network hydrogels. When they assembled the cell with Zn^2+^-CS/PAAm hydrogel electrolyte, the battery manifested superior electro-chemical performance with high specific capacity and retention. The Zn^2+^-CS/PAAm after self-healing hydrogel also exhibited excellent electro-chemical properties. Therefore, flexible hydrogel electrolytes are expected to replace conventional electrolytes and become novel electrolyte materials applied in more fields.

#### 4.2.2. Electrodes

**Figure 13:**
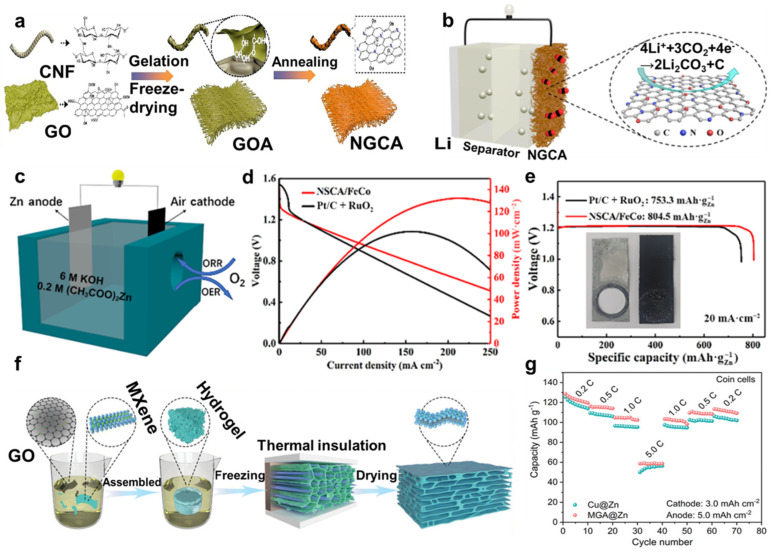
(**a**) Schematic diagram of the fabrication process of NGCA. (**b**) Schematic structure of Li−CO_2_ battery with NGCA cathode. (**c**) Illustration for the structure of liquid flow rechargeable ZAB structure. (**d**) Polarization curves and corresponding power density curves, and (**e**) specific capacity curves of Pt/C + RuO_2_ and NSCA/FeCo. (**f**) Schematic diagram of the synthesis process of MGA material. (**g**) Rate performance of the coin cells with different anode materials, reprinted with permission [121,122,124], copyright © 2023 Elsevier B.V., © 2023 Springer Nature, and © 2022 John Wiley & Sons, respectively.

Lithium-ion cells have attracted a great deal of attention in several years, and many materials with excellent performances are used as electrode materials to improve properties, especially aerogel. Alex et al. [120] prepared microporous carbon aerogel by the resorcinol–formaldehyde method through the ambient pressure drying route (at 1050 °C under Ar atmosphere), and it represented prominent capacity retention when it was applied as anode material in Li-ion batteries. Furthermore, lots of hybridized aerogels were used as electrode materials to make up for the shortcomings and challenges of batteries. For instance, Yu et al. [121] synthesized a self-standing and binder-free N-doped graphene carbon aerogel (NGCA) with certain oxygenic groups by a one-step method based on theoretical simulation (Figure 13a). The NGCA exhibited amicable characteristics of a highly efficient catalyst for Li-CO_2_ batteries as the cathodes, with elevated energy efficiency and prolonged cycle life (Figure 13b). Similarly, Zhang et al. [122] designed and prepared N and S co-doped carbon aerogel loaded with FeCo alloy nanoparticles (NSCA/FeCo), exhibiting outstanding bifunctional catalytic performance. Then, they assembled the rechargeable liquid flow ZABs with NSCA/FeCo as the air cathode (Figure 13c), showing excellent electro-chemical performance and bifunctional catalytic activity (Figure 13d,e), so NSCA/FeCo had promising potential for the rechargeable ZABs.

In addition to battery cathodes, aerogels can be applied to anodes as well. The modified cellulose aerogels were also applied for battery anodes. For instance, Yu et al. [123] designed free-standing electrodes using liquid metal anodes and fabricated a 3D network using eutectic Ga-In (EGaIn) nanoparticles (NPs) confined in carbon nanofibers and carbon nanotubes (CNF/CNT) to form the CNF/CNT@EGaIn anode, showing remarkable mechanical property, conductivity, and electro-chemical stability, offering a well-developed strategy for battery electrode production. Generally, aerogels can be regarded as amicable encapsulating substrates for the anode materials of batteries. For example, Zhou et al. [124] designed a foldable 3D MXene and graphene aerogel (MGA) with highly zincophilic properties (Figure 13f). Encapsulation of the Zn anode used MGA by one-step electrodeposition, and using the MGA@Zn anode and LiMn_2_O_4_ (LMO) cathode, assembled coin cells exhibited higher mechanical flexibility and cyclic stability (Figure 13g). This 3D encapsulating structure offered inspiration for the construction of battery anodes.

### 4.3. Supercapacitors

**Figure 14:**
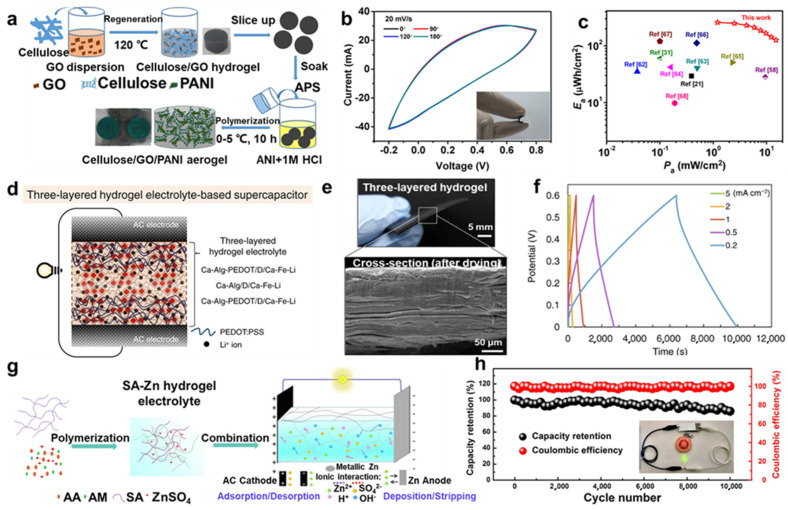
(**a**) Scheme of cellulose/GO/PANI aerogel production process. (**b**) CV curves of the device at different degree bending deformations. (**c**) Ragone plots and comparisons with other reported solid−state supercapacitors. (**d**) Structure of aqueous supercapacitor. (**e**) Cross−sectional SEM image of the three−layered hydrogel. (**f**) Galvanostatic charge–discharge (GCD) curves of aqueous supercapacitor at different current densities. (**g**) Scheme of the synthesis process and operation mechanism of the H−ZHS. (**h**) Cycling performance of H−ZHS at 5 A·g^−1^, inset as the lighting of green LED (start−up voltage 2 V) powered by H−ZHS, with permission [127,133,139], copyright © 2020 MDPI, © 2022 Springer Nature, and © 2020 Elsevier B.V., respectively.

Supercapacitors have attracted great attention as an important energy storage and transducer that can make up for the shortcomings of conventional energy storage cells. Supercapacitors employ hydrogel/aerogel-based electrodes owing to their outstanding electrical conductivity, high specific surface area, and mechanical properties [25,125,126]. For instance, Li et al. [127] synthesized free-standing cellulose/graphene oxide (GO) hybridized polyaniline (PANI) aerogel by in situ polymerization, coating PANI nanoclusters onto a cellulose/GO network (Figure 14a). The aerogel showed high conductivity, loading capacity, and electro-chemical reversibility, and it was promising for portable and lightweight electronics (Figure 14b,c). When it served as an electrode without any binder, it kept high flexibility and was bent to a large degree. And Crane et al. [128] synthesized transition metal dichalcogenide and carbon aerogel composites, since transition metal dichalcogenide converted to metallic phase demonstrated exceptional supercapacitor performance.

Hybridized hydrogels have been used as electrolytes [129,130,131]. For instance, Li et al. [132] designed electrolytes using gelatin/polyacrylamide (Gel/PAAm) double-network hybridized polypyrrole (PPy) nanoclusters to fabricate Gel/PAAm/PPy hydrogels with sandwich-like structures that are stretchable and conductive. These Gel/PAAm/PPy hydrogels were directly used in flexible all-in-one supercapacitors, providing energy storage and supply for other flexible electronics. In addition, Ji et al. [133] demonstrated a reconstructing process to synthesize alginate (Alg) hydrogels, adding some crosslinking ions (Ca^2+^, Ba^2+^, Al^3+^, or Fe^3+^) and conducting polymers to enhance their properties. Then, three-layered hydrogel electrolytes evolved from synthetic hydrogel, and activated carbon (AC) electrodes formed a mechanically robust aqueous supercapacitor (Figure 14d,e). The supercapacitor exhibited superior electro-chemical performance and stability (Figure 14f); thus, hydrogels had practical applications as solid gel electrolytes.

Furthermore, novel supercapacitors of aqueous zinc ion hybrid supercapacitors (ZHSCs), which combine the merits of Zn-ion batteries and supercapacitors, attracted great attention [134,135,136,137]. The studies on ZIHCS aimed to endow energy storage devices with superior properties and make up for the shortcomings of traditional storage materials. For example, Wang et al. [138] designed an organo-hydrogel electrolyte using poly(2-acrylamido-2-methylpropane-sulfonic acid) and polyacrylamide (PAMPS/PAAm) to fabricate a double-network matrix and binary solvent electrolyte, which exhibited high conductivity and flexibility in a wide range from −30 °C to 120 °C. The organo-hydrogel electrolytes for ZHSCs exhibited broad temperature adaptability with high capacitance retention and a long lifetime. Similarly, Han et al. [139] introduced acroleic acid (AA) and acrylamide (AM) monomers into a sodium alginate (SA) network with ZnSO_4_ electrolyte via free radical polymerization to form a double-network hydrogel electrolyte (SA-Zn hydrogel electrolyte) with amicable electro-chemical properties, used for a hybrid zinc-ion supercapacitor (H-ZHS) (Figure 14g). The H-ZHS exhibited high energy density, flexibility, and long cycling life (Figure 14h), showing a new idea of energy storage and flexible wearable electronics.

### 4.4. Photo-/Electro-Chemical Catalysis

The development of green, economic, and efficient catalysts is one of the key objectives among researchers and investigators. Aerogels have the advantages of high specific surface area and amicable porosity distributions, exposing more active sites and effective contact areas, which can be employed as carriers in catalysis. To date, aerogels are frequently used for heterogeneous catalysis [140], energy conversion, and degradation of hazardous species, especially in photocatalysis, electrocatalysis, and photoelectro-chemical fields.

**Figure 15:**
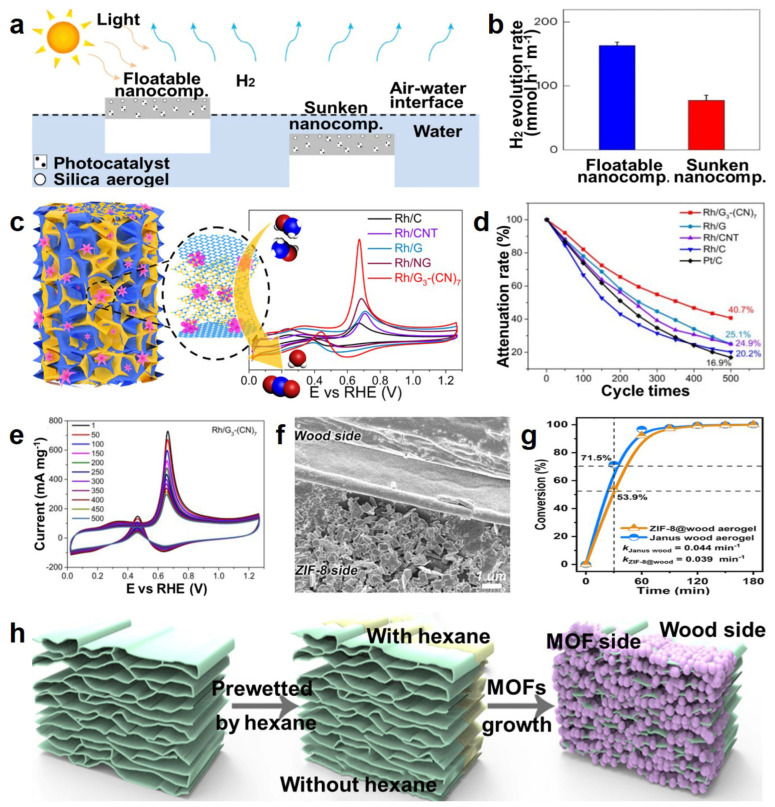
(**a**) Scheme of the photocatalytic HER process using floatable and sunken aerogel nanocomposites. (**b**) H_2_ evolution rate of the floatable and sunken aerogel nanocomposites. (**c**) Scheme of the 3D Rh/G−(CN) electrocatalyst structure and cyclic voltammogram (CV) profiles of the different electrodes in 1 mol L^–1^ KOH with 1 mol L^–1^ CH_3_OH mixed solution. (**d**) The methanol oxidation mass activities of various electrodes before and after cycling tests. (**e**) CV curves of the Rh/G_3_−(CN)_7_ electrode before and after 500 cycles. (**f**) SEM image of the Janus ZIF−8@wood aerogel. (**g**) The conversion of benzaldehyde using ZIF−8@wood aerogel and Janus ZIF−8@wood aerogel as catalysts. (**h**) Schematic illustration for the synthesis process of Janus MOFs@wood aerogel, reprinted with permission [141,143,144], copyright © 2023 Springer Nature and © 2021 American Chemical Society, respectively.

As for photocatalysts, Lee et al. [141] produced floatable nanocomposites composed of Pt/TiO_2_ cryogenic aerogel, single-atom copper/titanium oxide nanoparticles (Cu-SA/TiO_2_ NPs), and hydrophilic polyurethane-poly(propylene glycol)-sodium chloride (HPU-PPG-NaCl) gel, and the floatability was attributed to the Janus structure of two different surface properties, hydrophobic silica aerogel and hydrophilic gel layers. Based on the floatability, nanocomposites possessed unique features for photocatalysis, such as hydrogen production, and the floatable nanocomposites exhibited double the H_2_ evolution rate (163 mmol·h^–1^·m^–2^) compared with the sunken nanocomposites (H_2_ evolution rate of 77.2 mmol·h^–1^·m^–2^) (Figure 15a,b). Hereby, the floatable nanocomposite photocatalyst presented remarkable extensibility, durability, and photocatalytic activity, with high potential for the commercialization of solar hydrogen production. Similarly, Li et al. [142] designed a porous bismuth-based bromine oxides/cesium tungsten bronze (BiOBr/Cs_x_WO_3_)@SiO_2_ aerogel, showing high adsorption capability and photothermal catalytic synergies due to its ultrafine porosity, efficient self-heating photothermal conversion, and Cs_x_WO_3_/BiOBr heterojunction catalytic performance, offering a new idea for designing productive aerogel photocatalysts for environmental purification.

Regarding electrocatalysis, aerogel catalysts play essential roles in energy conversion and storage devices. For example, Du et al. [143] fabricated flower-like 3D hybrid aerogels using rhodium crystal, graphene, and graphitic carbon nitride nanolayers (Rh/G-CN) via a bottom-up synthetic strategy. The Rh/G-CN aerogel exhibited excellent conductivity, catalytic properties with a large electrochemically active surface area, high current density, a long lifespan for methanol electro-oxidation, and long-term stability for the methanol fuel cells (Figure 15c–e), which were attributed to the network structure with abundant channels and macropores providing a large number of catalytic active sites. Additionally, Wu et al. [144] designed metal–organic frameworks (MOFs) loaded with Janus wood aerogel (Janus MOFs@wood aerogels), possessing asymmetric structure and wettability via the liquid deposition method (Figure 15f,h). This aerogel maintained the layered structure and demonstrated hierarchical porosity along with good mechanical compressibility, showing better catalytic properties than ZIF-8 crystalline powders in the Knoevenagel reaction. And the Janus MOFs@wood aerogel presented superior catalytic performance by Le Châtelier’s principle compared with MOFs@wood aerogel (Figure 15g). This investigation provided a novel path for the development of 3D hybrid electrocatalysts with special structures to apply to more fields.

### 4.5. Biomedical Applications

**Figure 16:**
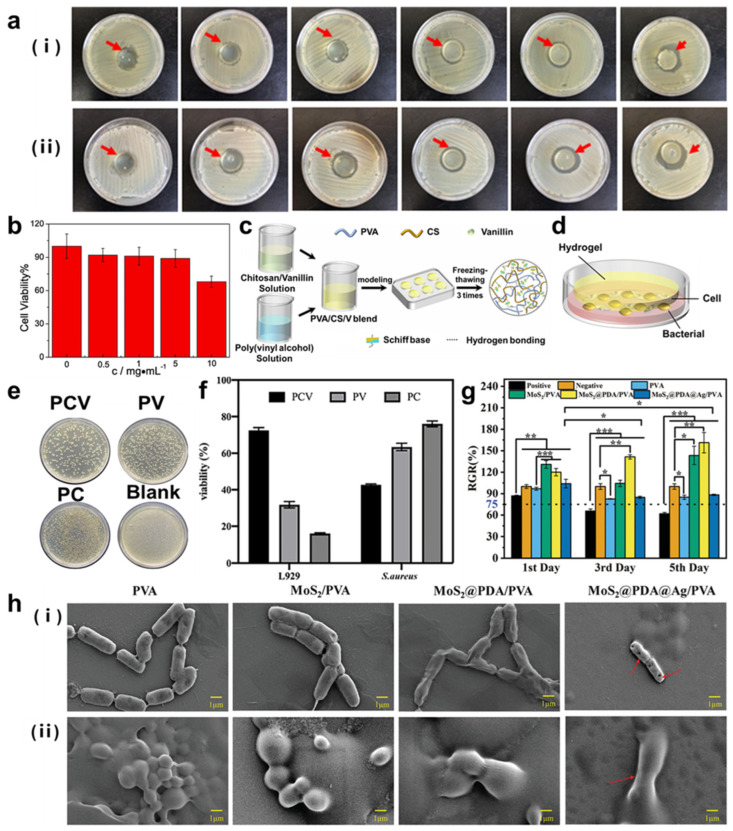
(**a**) Antibacterial effect of different concentrations of PHMG hydrogels on *E. coli* (i) and *S. aureus* (ii). From left to right, the concentration of PHMG increases, 0%, 0.005%, 0.01%, 0.05%, 0.1%, and 0.5%, respectively. (**b**) Cell counting kit−8 (CCK−8) assays of the PVA/PAAm IPN hydrogel against human embryonic kidney cells. (**c**) Schematic illustration of the synthetic process of PCV hydrogel. (**d**) Simulation model of antibacterial experiment in vitro. The in vitro antibacterial experiment of (**e**) the distribution of *S. aureus* colony in culture dish after coating for 24 h and (**f**) the relative activity of L929 cells and *S. aureus*. (**g**) The in vitro cytocompatibility experiment of PVA−based hydrogels examined against L929 cells after incubation for 1, 3, and 5 days via MTT assay. * *p* < 0.05, ** *p* < 0.01, *** *p* < 0.001. (**h**) SEM images of (i) *E. coli* and (ii) *S. aureus* captured after irradiation with NIR light at 808 nm for 15 min, reprinted with permission [145,146,147], copyright © 2021−2022 Elsevier B.V. and © 2021 John Wiley & Sons, respectively.

Since the antibacterial medium plays a necessary role in contagions and inflammatory infections, a growing number of investigations on hydrogels or aerogels have drawn curiosities for biomedical fields, especially antibacterial systems. For example, Li et al. [145] fabricated PVA/PAAm IPN hydrogels and introduced an electropositive polymer antibacterial agent, polyhexamethylene guanidine (PHMG), showing excellent antibacterial properties. They evaluated both the Gram-negative and Gram-positive bacteria using *E. coli* and *S. aureus*, substantiating that the hydrogel containing PHMG had a significant inhibitory effect on the growth of Gram-positive and Gram-negative bacteria (Figure 16a). Furthermore, the cytotoxicity of PVA/PAAm IPN hydrogels was tested by CCK8 assay, and results showed that PVA/PAAm IPN hydrogels had low cytotoxicity (Figure 16b). Similarly, vanillin was introduced into polyvinyl alcohol-chitosan hydrogel to form a PVA/CS/V (PCV) hybrid (Figure 16c) [146], and PCV hydrogels in each group showed an excellent antibacterial effect for *E. coli* and *S. aureus*, and the size of the antibacterial ring increased with the increase in vanillin concentration. An in vitro antibacterial model was developed to explore the potential application of PCV hydrogels (Figure 16d). According to the results of the simulation experiment, PCV hydrogels prohibited the growth of bacteria and promoted the cell viability of the L929 cell line (Figure 16e,f), which was prominent for the application of wound dressing.

Further applications such as wound healing [148,149] usually adopt nanomaterials [150,151] and polymers [152,153] to promote mechanical properties and allow performance with amicable biocompatibility and low toxicity, such as CNT, tannic acid, PDA, etc. For example, Yan et al. [147] prepared a PVA hybrid hydrogel by incorporating PVA with molybdenum disulfide, polydopamine, and silver (MoS_2_@PDA@Ag/PVA), with excellent photothermal therapy (PTT) and photodynamic therapy (PDT) effects for high antibacterial activity against *S. aureus* and *E. coli*. The introduction of MoS_2_, PDA, and Ag nanoparticles improved mechanical property, photocatalytic performance, and PTT and PDT effects for antibacterial activity. Based on the synergistic effect between photothermal and photodynamic properties, the hydrogel exhibited a superior antibacterial effect under near-infrared (NIR) light irradiation (Figure 16h). Furthermore, the cytocompatibility of PVA-based hydrogel was tested in vitro (Figure 16g), according to the 3-(4,5-dimethylthiazol-2-yl)-2,5-diphenyltetrazolium bromide (MTT) assay. Thus, the biomedical hydrogel is expected to be applied to wound healing.

### 4.6. Thermal Insulation

**Figure 17:**
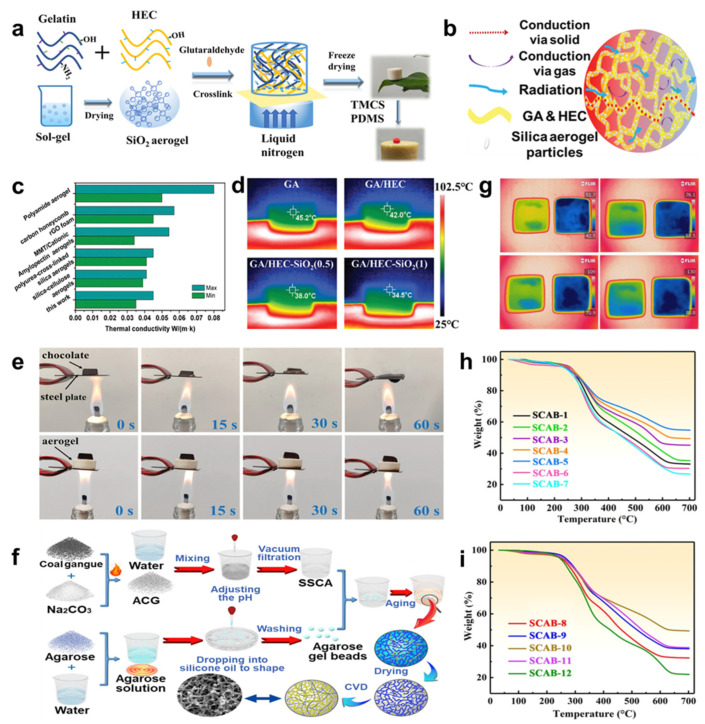
(**a**) Scheme of GA/HEC-SiO_2_ aerogels’ production process. (**b**) The heat transfer process of composite aerogels. (**c**) Thermal conductivity comparison of GA/HEC-SiO_2_ aerogel with other aerogels. (**d**) The pseudo-color thermal images of different aerogels settled on continuous heating at 100 °C platform. (**e**) Thermal insulation and stability tests of the GA/HEC-SiO_2_ aerogel. (**f**) Scheme of the SCAB fabrication process. (**g**) Forward-looking infrared (FLIR) images of the putty block without and with SCABs heating bottom plates at different temperatures. (The unit of temperature scale bar on right is °C). (**h**,**i**) Thermal stability characterization of SCABs using the thermogravimetric analysis profile, reprinted with permission [28,155], copyright © 2022 MDPI and © 2021 John Wiley & Sons, respectively.

The introduction of thermal insulation is a way to solve the problem of energy consumption. Aerogels possessing high porosity, low thermal conductivity, and a tiny density [154] are considered excellent candidates for thermal insulation, such as metal oxide and cellulose aerogels. For example, Peng et al. [155] successfully constructed the gelatin (GA)/hydroxyethyl cellulose (HEC)-SiO_2_ aerogel via the sol–gel freeze-drying method (Figure 17a), showing excellent mechanical strength and superior thermal insulation properties with long-term stability (Figure 17b–e), owing to the special distribution of SiO_2_ particles inside walls of aerogel pores and generating multiple hydrogen bonds as the crosslinking bridges among these matrix materials. Similarly, adding additives to oxide aerogels to form hybridized 3D networks is a useful and popular technique. Gu et al. [28] fabricated SiO_2_–Al_2_O_3_/agarose hybrid aerogel beads (SCABs) by simple chemical vapor deposition of trimethylchlorosilane (Figure 17f), maintaining high hydrophobicity and mechanical and thermal insulation properties with persistent stability owing to the unique interpenetrating network architectures (Figure 17g–i), indicating that they could accommodate further domains and broaden prospective functionalities to meet the requirements of more applications.

## 5. Summary and Prospects

This work demonstrates illustrations of the properties, structures, and applications of hydrogels and aerogels. Photo-/electro-chemical and energy-related fields such as sensors, batteries, supercapacitors, and biomedical applications demand the versatile functionalities and fine-tuned properties of modified hybrid gels. When they are used for the electrolyte and electrode materials of energy devices, they have the potential to replace conventional electrolyte and electrode materials. Increasing studies devoted to applying gels for recording physiological health signals, such as electroencephalography, electrocardiogram, electromyography, and electrooculogram, exhibit valuable potential for photo-/electro-chemical and biomedical applications.

Hydrogels can immobilize catalysts and enzymes in a 3D network by physical or chemical methods to achieve catalytic and biocatalytic reactions, respectively. Additionally, hydrogels can also be used for catalytic hydrogenation, oxidation, and esterification. However, the catalytic efficiency of the hydrogel may be limited by mass transfer since the reactants and products need to diffuse in the hydrogel network. And hydrogels can dehydrate within a certain time, leading to unsatisfactory performance and a short lifetime during the operation process. In addition, the stability and reusability of hydrogels also need to be further improved. For future research prospects, novel hydrogel materials are demanded with higher porosity, better mass transfer properties, and improved durability and performance. Typically, combining hydrogels with other materials (e.g., polymers, nanoparticles, quantum dots, etc.) to form hybridized composites with higher performance and stability is also a research hotspot. Incorporation of PDMS as substrate material to form composite elastomers is also promising for photo-/electro-chemical and flexible energy-related devices, improving long-term stability and superior properties.

Aerogels possess a specific ultrafine 3D porous network that can facilitate the dispersion of active components and thus present a significant impact on catalytic processes and accommodate energy-related fields. The activity and selectivity of aerogels are much higher than those of conventional catalysts, and the active components can be very evenly dispersed on the support. Meanwhile, aerogels demonstrate excellent thermal stability, which effectively reduces the occurrence of side reactions. Organic and inorganic composite aerogels have been applied for thermal insulation, presenting superior performances and solving problems of energy consumption. However, the current preparation cost of aerogel is high, which limits its promotion in large-scale industrial applications to date. And the strength and stability of aerogels require further improvement. Future research directions include developing low-cost preparation methods, improving the strength and stability of aerogels, and exploring more applications of aerogels and hydrogels in expanded photo-/electro-chemical and energy-related fields.

## Data Availability

Data are contained within the article.
